# Evaluation of regional differences, age, and sex variations in hematological parameters among adolescents in Sudan: a multicenter community-based cross-sectional study

**DOI:** 10.3389/fped.2026.1928522

**Published:** 2026-08-20

**Authors:** Tasabeh M. Eltayb, Ahmed A. Hassan, Abdullah Al-Nafeesah, Ashwaq AlEed, Saeed M. Omer, Ishag Adam

**Affiliations:** 1 Department of Family and Community Medicine, College of Medicine, Najran University, Najran, Saudi Arabia; 2 Faculty of Medicine, University of Khartoum, Khartoum, Sudan; 3 Department of Pediatrics, College of Medicine, Qassim University, Khartoum, Saudi Arabia; 4 Department of Medcine, Gadarif University, El-Gadarif, Sudan; 5 Department of Obstetrics and Gynecology, College of Medicine, Qassim University, Buraydah, Saudi Arabia

**Keywords:** age, ethnicity, gender, hemoglobin, platelets, red cell distribution width

## Abstract

**Background:**

Recently, greater attention has been paid to the influence of regional differences, age, and sex on hematological parameters, particularly among adolescents. No such data are available in Sudan. The current study aimed to investigate the associations between regional differences, age, sex, and hematological parameters [hemoglobin, white blood cell [WBC], red cell distribution width [RDW], and platelets] among adolescents in northern and eastern Sudan.

**Methods:**

A comparative community-based cross-sectional study was conducted in northern and eastern Sudan. The adolescents’ sociodemographic data were collected via a questionnaire. The studied hematological parameters were measured using an automated hematology analyzer. Multiple linear regression analysis was performed.

**Results:**

A total of 738 adolescents participated in the study, comprising 356 from northern Sudan and 382 from eastern Sudan, with 44.0% males and 56.0% females. The median age was 14.8 years, and the hematological parameters were as follows: hemoglobin 12.9 g/dL, WBC 6.0 × 10^9^/L, red blood cells 4.7 × 10^6^/mm^3^, platelets 348.0 × 10^9^/L, and RDW 13.7%. Hemoglobin and RDW levels were significantly higher among adolescents in northern Sudan than in eastern Sudan. Conversely, WBC and platelet counts were significantly higher in eastern Sudan. Multivariate linear regression analysis revealed that increasing age was positively associated with hemoglobin but negatively associated with platelet count. Being female was positively associated with WBC, RDW, and platelets but negatively associated with hemoglobin levels. Living in eastern Sudan was positively associated with WBC and platelets but negatively associated with hemoglobin and RDW.

**Conclusion:**

The study identified notable regional variations in hematological parameters among adolescents in Sudan, with northern Sudan showing higher hemoglobin and RDW levels, whereas eastern Sudan had increased WBC and platelet counts. Further research is recommended to establish age-, regional differences-, and gender-adjusted cutoff values for hematological parameters in clinical practice to improve the diagnosis and prognosis of hematological disorders among Sudanese adolescents.

## Introduction

Hematological parameters, including hemoglobin, white blood cell (WBC) count, red cell distribution width (RDW), and platelet count, are vital indicators of health status in adolescents (1–3). These parameters can vary significantly due to intrinsic factors such as regional differences, age, and sex, which can influence the development of hematological disorders and the general health of this population (1–3). The interaction between regional differences, age, and gender further complicates the understanding of hematological parameters in adolescents. For instance, differences in dietary practices among different regions can influence iron intake and, consequently, hemoglobin levels (4). Additionally, cultural beliefs about health and nutrition may lead to varying dietary habits, which can further impact blood parameters across different ethnicities. Research has demonstrated that adolescents from various regional and cultural backgrounds may have distinct nutritional profiles, which can contribute to variations in hemoglobin, WBC, RDW, and platelet counts. It has been reported that regional differences and cultural factors significantly influence dietary choices, leading to disparities in iron and vitamin intake, both of which are essential for maintaining healthy hematological parameters (4). Furthermore, the combined effects of age and sex on hematological parameters can lead to complex relationships (3, 5). For example, as adolescents age, the impact of sex on hemoglobin levels becomes more pronounced, with males typically experiencing a greater increase in hemoglobin levels due to hormonal changes during puberty (5). Conversely, females may experience declines in hemoglobin levels due to menstruation, which can also vary by regional differences depending on cultural practices surrounding menstruation and health (6).

Therefore, the influence of regional differences, age, and sex on hematological parameters such as hemoglobin, WBC, RDW, and platelets in adolescents is multifaceted and significant. Consequently, it necessitates setting region-specific hematological parameter reference intervals, especially in Africa, where multiethnicity exists (3, 7–10). Sudan is characterized by diverse regional differences among groups (11)each with a unique regional background that can affect hematological parameters. Research has shown that ethnic differences contribute significantly to variations in hemoglobin levels and the prevalence of hematological disorders. For instance, sickle cell disease is more prevalent among specific African populations, leading to significant differences in hemoglobin concentrations and related health outcomes (12). Understanding these influences is critical for healthcare professionals to provide accurate diagnoses and effective treatment plans tailored to adolescents' individual needs. Establishing age- and regional differences-specific reference ranges for hematological parameters can improve clinical practice. Thus, the current study aimed to investigate the association between regional differences, age, sex, and hematological parameters, including hemoglobin, WBC, RDW, and platelets, in apparently healthy adolescents in northern (Almatamah, River Nile State) and eastern (Gadarif) Sudan. These two regions have entirely different populations, are inhabited by distinct tribes, and are located at least 600 kilometers apart. Our previous study showed that glycated hemoglobin (HbA1c) levels are influenced by regional differences and age in the two studied regions (eastern and northern Sudan) (13).

## Materials and methods

### Study areas

Details of the study areas have been outlined in our previous work. In summary, a comparative community-based cross-sectional study was conducted in northern Sudan from June to September 2022, followed by another survey in eastern Sudan from August to October 2023. The research took place in River Nile State, northern Sudan, and Gadarif State, eastern Sudan, both of which are among Sudan's 18 states. According to the 2008 census, the populations of River Nile State and Gadarif State were 1,120,441 and 1,400,000 people, respectively (14). River Nile State is divided into seven localities, the smallest administrative units in Sudan. Among these, the Almatamah Locality was selected for study because its population primarily descends from the Jaalain tribe. Almatamah is located approximately 100 kilometers from Khartoum, the capital of Sudan. In contrast, Gadarif State is characterized by a multi-ethnic society, encompassing various Sudanese tribes from different regions (15). Gadarif State is situated in eastern Sudan, bordering Ethiopia, with Gadarif city as its capital. It comprises eleven localities and boasts vast agricultural land, hosting some of Sudan's largest rain-fed agrarian projects. Gadarif locality was selected from the eleven, and an appropriate sample size was recruited. More information about the study areas was given in our previously published data (16, 17).

### Study population and design

An appropriate sample size was determined for each selected locality based on population density to achieve the desired total sample size. Households were randomly selected using the lottery method. The number of selected households was proportionate to the populations in the area. One adolescent from each household was randomly selected (lottery method). When there was no adolescent in the selected household, or they refused to participate, the next household was selected. To standardize the data collection process and minimize interpersonal biases, the investigators trained five medical officers in data collection methods to gather data from the two regions.

### Definitions

This study followed WHO's definition of adolescents (aged 10 to 19 years). According to the WHO estimation, in Sudan, more than 20% of the population is adolescent (18). Hemoglobin levels were categorized according to the WHO guidelines, with participants classified as anemic if their hemoglobin was less than 12.0 g/dL and non-anemic if it was 12.0 g/dL or higher (19). Participants with known hemoglobinopathies were excluded from the study.

### Inclusion criteria

Sudanese adolescents (both males and females) aged 10 to 19 years living in a household were selected using a lottery method. Participants were required to be healthy individuals with no known current illnesses. An “apparently healthy status” lacking physical signs of disease during a baseline evaluation (vital signs, physical exam, symptom checklist), with explicit exclusion criteria (e.g., active infections, diabetes, asthma, renal and thyroid disease, congenital disorders).

### Exclusion criteria

Participants aged less than 10 years or over 19 years, those with existing hemoglobinopathies, pregnant women, individuals with poor cognitive function, and severely ill patients were excluded from this study.

### Data collection

The questionnaire was developed based on previous similar studies (3, 7–10) and was used to collect data on sociodemographic characteristics, including age (years), gender (male or female), location (eastern or northern Sudan), and smoking/tobacco use (yes or no).

### Sample collection and processing

A 3–5 mL blood sample was collected from all participants under aseptic conditions for testing of hematological parameters. First, 1 mL was taken from each sample to perform the complete blood count (CBC) parameters per the manufacturer's instructions (Sysmex KX-21, Japan). As described in our previous work, an automated hematology analyzer was used to perform CBC, including hemoglobin, WBC, platelets, RDW, mean corpuscular volume (MCV), mean platelet volume (MPV), mean corpuscular hemoglobin concentration (MCHC), and other (20). Second, the remaining sample (4 mL) of blood was centrifuged and kept at −20 °C until processed in the laboratory for CRP, as described in our previously published work (21).

### Sample size calculation

The desired sample size was computed using the OpenEpi Menu (22). A total sample size of 738 participants was calculated, with a 1.1:1 ratio between Gadarif State and River Nile State based on their respective populations. We assumed a difference of 0.2 in mean hemoglobin levels between participants in Gadarif State and those in River Nile State (12.8 vs. 13.0 g/dL). This sample size was determined to detect a 5% difference at *α* = 0.05 with 80% power.

### Statistical analysis

The collected data were entered into IBM Statistical Product and Service Solutions (SPSS) for Windows (version 22.0; SPSS Inc., New York, NY) for analysis. Continuous variables, including age and hematological parameters (hemoglobin, WBC, RDW, MPV, and MCH), were assessed for normality using the Kolmogorov–Smirnov test and found to be non-normally distributed. As a result, these variables were reported as medians [interquartile ranges (IQRs)]. The levels of hematological parameters (WBC, hemoglobin, MPV) and CRP were compared between the two locations using the Mann–Whitney non-parametric test. To determine the influence of certain factors, namely regional differences, age, sex, and smoking/tobacco use (independent variables), on the studied hematological variables (dependent variables), significant hematological parameters (hemoglobin, WBC, RDW, platelets) with *p*-values < 0.05 in the Mann–Whitney test were analyzed using univariate linear regression. Additionally, multivariate linear regression was conducted for each significant hematological parameter (hemoglobin, WBC, RDW, platelets) as dependent variables, controlling for potential confounding factors. Other hematological variables, such as hematocrit (HCT), MCV, and MCHC, which were significant between the two locations, were not further analyzed by linear regression, as they depend on hemoglobin and platelets for their calculation (23). Multicollinearity (variance inflation factor < 4) was checked for but not detected. The results included adjusted regression coefficients (*β* coefficients) with 95% confidence intervals (CIs), and a *p*-value of <0.05 was considered statistically significant.

## Results

**Figure 1:**
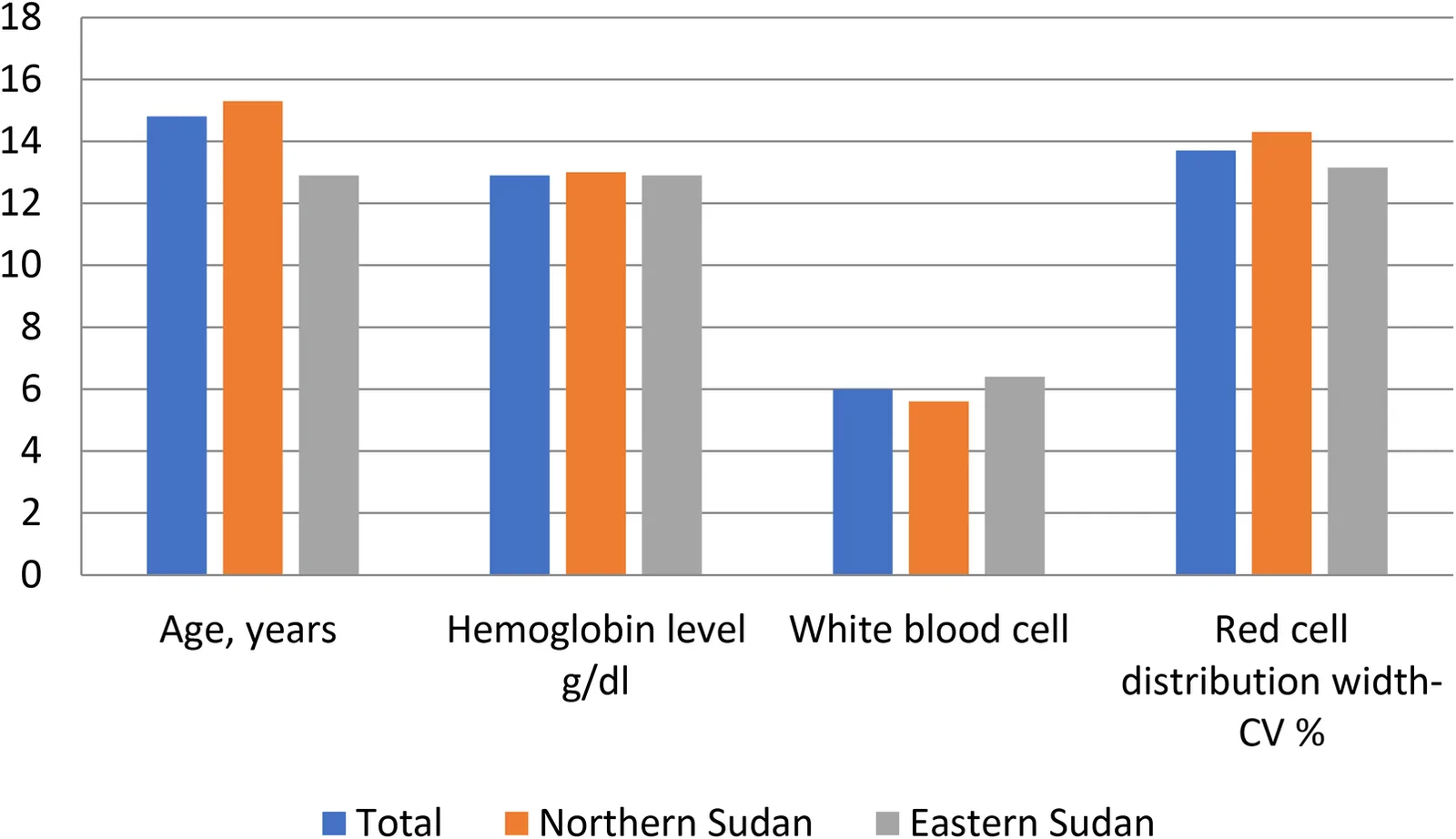
Shows the median age, hemoglobin, white blood cell, and red cell distribution width-CV in adolescents between northern and eastern Sudan (*n* = 738).

**Figure 2:**
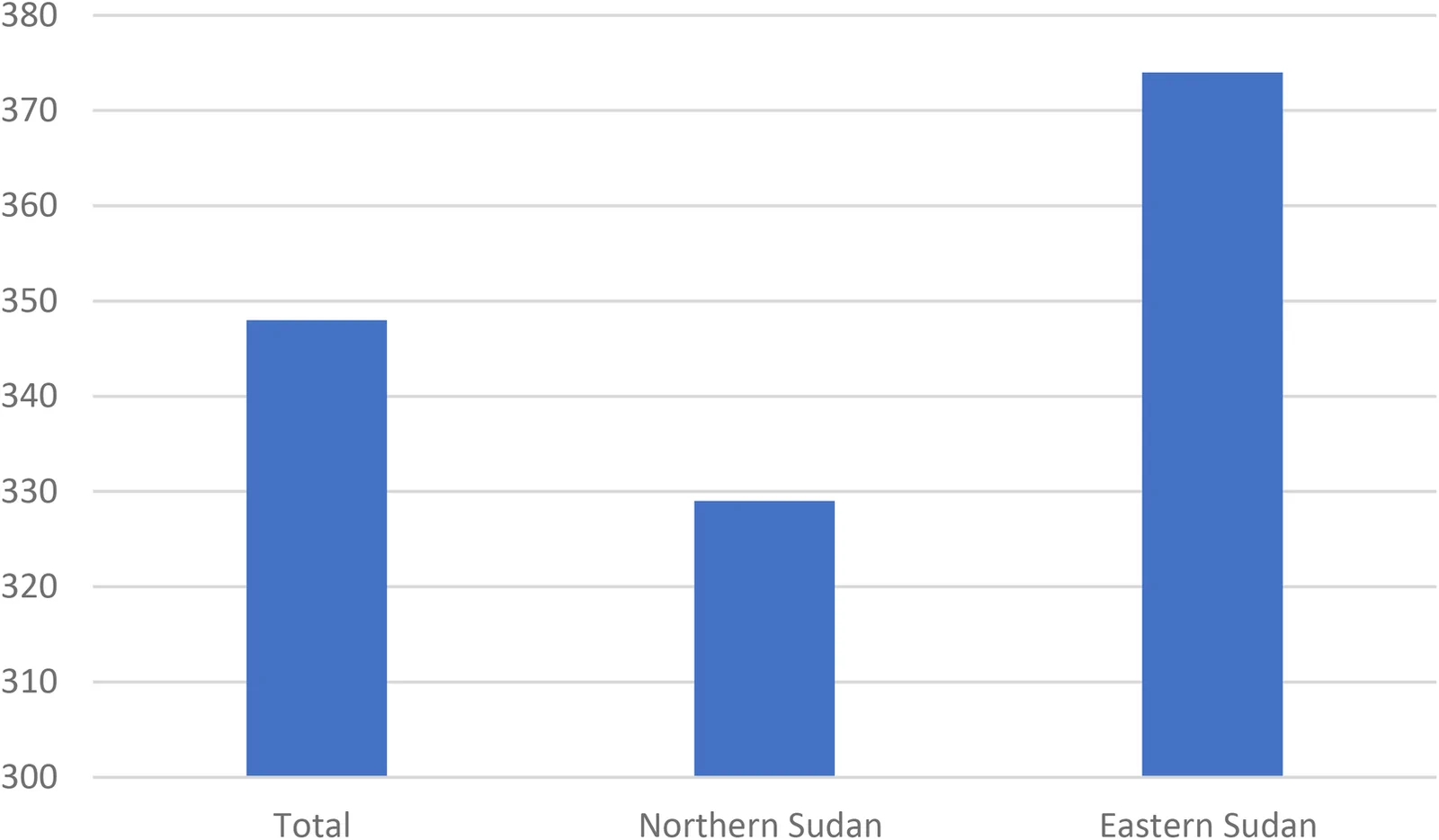
Shows the median platelet count in adolescents between northern and eastern Sudan (*n* = 738).

A total of 738 adolescents participated in the study, with 356 from northern Sudan and 382 from eastern Sudan. The sample comprised 325 males (44.0%) and 413 females (56.0%). The median (IQR) values for age, hemoglobin, WBC, RBC, platelets, and RDW were 14.8 (13.1–16.2) years, 12.9 (12.1–13.7) g/dL, 6.0 (4.9–7.5) × 10^9^/L, 4.7 (4.45–4.98) × 10^6^/mm^3^, 348.0 (293.0–411.0) × 10^9^/L, and 13.7 (13.0–14.6) %, respectively. No significant differences in sex, HCT, RBC, or CRP levels were observed between adolescents from northern and eastern Sudan. The median (IQR) hemoglobin and RDW levels, as well as platelets, were significantly higher in north Sudan compared to eastern Sudan [13.0 (12.3–14.0) g/dL vs. 12.9 (12.0–13.6) g/dL, *P* = 0.006] and [14.3 (13.7–15.1) vs. 13.15 (12.67–13.8) %, *P* < 0.001], respectively. Conversely, WBC and platelets were significantly higher in eastern Sudan compared to northern Sudan [6.4 (5.2–7.7) vs. 5.6 (4.6–7.1), 10^9^/L, *P* < 0.001] and [374.0 (309.0–436.0) vs. 329.0 (285.0–382.0) × 10^9^/L, *P* < 0.001], respectively (Table 1, Figures 1, 2). Among the 37 tobacco users, more adolescents were smoking in northern Sudan than in eastern Sudan (29 vs. 8). Of the 222 adolescents identified as anemic, more were from eastern Sudan than from northern Sudan (143 vs. 79), as shown in Table 1.

**Table 1:** Characteristics of the studied adolescents (males and females) in northern and eastern Sudan (*n* = 738).

Variable		Total (number = 738)	Northern Sudan (number = 356)	Eastern Sudan (number = 382)	*P* value
Median (interquartile range)					
Age, years		14.8 (13.1‒16.2)	15.3 (14.0‒16.4)	12.9 (12.0‒13.6)	<0.001
Hemoglobin level for both sexes, g/dL		12.9 (12.1‒13.7)	13.0 (12.3‒14.0)	12.9 (12.0‒13.6)	0.006
Hemoglobin for all females, g/dL		12.7 (11.9‒13.3)	12.7 (12.0‒13.2)	13.0 (12.3‒13.8)	0.628
Hemoglobin for all males, g/dL		13.4 (12.6‒14.3)	13.9 (13.0‒14.6)	12.7 (11.9‒13.4)	<0.001
White blood cell, ×10^9^/L		6.0 (4.9‒7.5)	5.6 (4.6‒7.1)	6.4 (5.2‒7.7)	<0.001
Red blood cell count, ×10^6^/mm^3^		4.7 (4.45‒4.98)	4.68 (4.43‒4.96)	4.73 (4.50‒5.0	0.074
Hematocrit, %		38.2 (36.4‒40.3)	38.2 (36.4‒40.4)	38.3 (36.3‒40.2)	0.825
Mean corpuscular volume, fL		81.6 (78.47‒84.8)	82.1 (79.1‒85.4)	81.3 (77.8‒84.1)	0.004
Mean corpuscular hemoglobin, Pg		27.6 (26.3‒28.8)	27.9 (26.7‒29.0)	27.3 (26.0‒28.4)	<0.001
Mean corpuscular hemoglobin concentration, g/dL		33.7 (33.0‒34.4)	33.8 (33.2‒34.5)	33.6 (32.6‒34.3)	<0.001
Red cell distribution width, %		13.7 (13.0‒14.6)	14.3 (13.7‒15.1)	13.15 (12.67‒13.8)	<0.001
Platelet count,10^9^/L		348.0 (293.0‒411.0)	329.0 (285.0‒382.0)	374.0 (309.0‒436.0)	<0.001
Mean platelet volume, %		9.0 (8.4‒9.8)	8.4 (8.0‒8.9)	9.7 (9.1‒10.4)	0.001
Platelet distribution width%		14.8 (11.4‒15.4)	15.3 (15.2‒15.6)	11.4 (10.6‒12.8)	0.001
Frequency (proportion)					
Sex	Male	325 (44.0)	148 (41.6)	177 (46.3)	0.193
	Female	413 (56.0)	208 (58.4)	205 (53.7)	
Smoking/tobacco use	No	701 (95.0)	327 (91.9)	374 (97.9)	<0.001
	Yes	37 (5.0)	29 (8.1)	8 (2.1)	
Anemia	No	516 (69.9)	277 (77.8)	239(62.6)	<0.001
	Yes	222(30.1)	79(22.2)	143(37.4)	

In the univariate linear regression analysis of all participants, age (coefficient = 0.059, *P* = 0.014) and smoking/tobacco use (coefficient = 0.482, *P* = 0.040) were positively associated with hemoglobin levels, while being female (coefficient = −0.887, *P* < 0.001) and living in eastern Sudan (coefficient = −0.276, *P* = 0.007) were negatively associated with hemoglobin levels. For WBC, being female (coefficient = 0.436, *P* = 0.023) and residing in eastern Sudan (coefficient = 0.392, *P* = 0.040) were positively associated with WBC. No associations with WBC were found for age (coefficient = −0.028, *P* = 0.529) or smoking/tobacco use (coefficient = −0.359, *P* = 0.412). Age (coefficient = 0.114, *P* < 0.001) and being female (coefficient = 0.317, *P* = 0.012) were positively associated with RDW, whereas living in eastern Sudan (coefficient = 1.162, *P* < 0.001) was negatively associated with RDW. Smoking/tobacco use did not correlate with RDW (coefficient = 0.399, *P* = 0.164). Regarding platelets, being female (coefficient = 28.718, *P* < 0.001) and residing in eastern Sudan (coefficient = 35.633, *P* < 0.001) were positively associated, whereas age (coefficient = −5.936, *P* = 0.001) was negatively associated. Smoking/tobacco use was not associated with platelets (coefficient = −31.352, *P* = 0.062), as summarized in Table 2.

**Table 2:** Univariate linear regression analysis of factors associated with hematological parameters among adolescents (males and females) in northern and eastern Sudan (*n* = 738).

Variable	Hemoglobin level g/dL		White blood cell, 10^9^/L		Red cell distribution width %		Platelets, 10^9^/L	
	Coefficient (standard error)	*P*	Coefficient (standard error)	*P*	Coefficient (standard error)	*P*	Coefficient (standard error)	*P*
Age, years	0.059 (0.024)	0.014	−0.028 (0.045)	0.529	0.114 (0.029)	<0.001	−5.936 (1.70)	0.001
Gender (female vs. male)	−0.887 (0.098)	<0.001	0.436 (0.192)	0.023	0.317 (0.125)	0.012	28.718 (7.323)	<0.001
Location (eastern vs. northern)	−0.276 (0.102)	0.007	0.392 (0.191)	0.040	−1.162 (0.118)	<0.001	35.633 (7.233)	<0.001
Smoking/tobacco use (user vs. non-user)	0.482 (0.234)	0.040	−0.359 (0.437)	0.412	0.399 (0.286)	0.164	−31.352 (16.793)	0.062

In the multivariate linear regression analysis, age was positively associated with hemoglobin levels (coefficient = 0.068, *P* = 0.004) but negatively associated with platelets (coefficient = −4.93, *P* = 0.005). Age has no association with WBC (coefficient = −0.017, *P* = 0.714) or RDW (coefficient = 0.046, *P* = 0.108). Being female was positively associated with WBC (coefficient = 0.459, *P* = 0.018), RDW (coefficient = 0.242, *P* = 0.043), and platelets (coefficient = 32.647, *P* < 0.001), and negatively associated with hemoglobin levels (coefficient = −0.929, *P* < 0.001). Residing in eastern Sudan was positively associated with WBC (coefficient = 0.389, *P* = 0.049) and platelets (coefficient = 31.647, *P* < 0.001), and negatively associated with hemoglobin (coefficient = −0.242, *P* = 0.01) and RDW (coefficient = −1.102, *P* < 0.001). Smoking/tobacco use was not associated with the hematological parameters of hemoglobin (coefficient = 0.129, *P* = 0.566), WBC (coefficient = −0.123, *P* = 0.782), RDW (coefficient = 0.029, *P* = 0.916), or platelets (coefficient = −7.98, *P* = 0.632), (confounding factor), as shown in Table 3.

**Table 3:** Multivariate linear regression analysis of factors associated with hematological parameters among adolescents (males and females) in northern and eastern Sudan (*n* = 738).

Variable	Hemoglobin level g/dL		White blood cell		Red cell distribution width %		Platelets	
	Coefficient (standard error)	*P*	Coefficient (standard error)	*P*	Coefficient (standard error)	*P*	Coefficient (standard error)	*P*
Age, years	0.068 (0.023)	0.004	−0.017 (0.046)	0.714	0.046 (0.029)	0.108	−4.93 (1.73)	0.005
Gender (female vs. male.	−0.929 (0.098)	<0.001	0.459 (0.194)	0.018	0.242 (0.120)	0.043	32.44 (7.26)	<0.001
Location (eastern vs. northern)	−0.242 (0.100)	0.015	0.389 (0.197)	0.049	−1.102 (0.122)	<0.001	31.647 (7.377)	<0.001
Smoking/tobacco use (use vs. non-use)	0.129 (0.225)	0.566	−0.123 (0.445)	0.782	0.029 (0.275)	0.916	−7.98 (16.67)	0.632

## Discussion

This study recognized the influence of regional differences, age, and gender on hematological parameters, including hemoglobin, WBC, platelets, and RDW, among adolescents in Sudan. Adolescents in northern Sudan had higher hemoglobin and RDW levels, whereas those in eastern Sudan had increased WBC and platelet counts. Likewise, several studies found differences in hematological parameters such as hemoglobin, WBC, platelets, and RDW in adolescents (2, 3, 24) and adults (7, 9, 10, 25, 26). For example, in India, a national sample of 8,087 healthy children and adolescents aged 1–19 years called for a re-examination of the WHO hemoglobin cutoffs to define anemia across different regions and ethnic populations (3). According to the researchers, regional differences significantly influence hematological parameters such as hemoglobin levels, WBC count, RDW, and platelet count in adolescents, owing to genetic, environmental, and sociocultural factors. Genetic predispositions among different ethnic groups can lead to variations in hematological parameters (12, 27). For instance, certain populations may have inherited traits that affect hemoglobin production, including a higher prevalence of conditions such as sickle cell disease and thalassemia, which are more common in individuals of African and Mediterranean descent (12, 27). These regional differences can result in lower hemoglobin levels in some groups compared to others. Regional differences in dietary habits can affect iron and vitamin intake, which is crucial for maintaining healthy hematological profiles. However, the median difference in hemoglobin between regions is relatively small, despite being statistically significant. Thus, the observed differences are unlikely to influence clinical decision-making. Moreover, the median hemoglobin in both regions and in males and females is above the WHO cut-off for anemia (28).

Adolescents from regional backgrounds with limited access to nutritious foods may exhibit lower hemoglobin levels and altered RDW values due to nutrient deficiencies. For example, a study highlighted positive associations between body mass index (BMI) and various hematological parameters, indicating that nutritional status influences hematological health across different regions/ethnic groups (29). Socioeconomic status often correlates with access to healthcare, nutrition, and overall health outcomes. In some regions, barriers to healthcare lead to undiagnosed or untreated hematological disorders. Research has shown that metabolic syndromes, which can affect hematological parameters, are more prevalent in certain ethnic groups, underscoring the importance of accounting for regional differences when assessing risks of conditions such as obesity and related hematological changes (30, 31). Cultural beliefs and practices regarding diet and health can also influence hematological parameters. Therefore, the interplay of genetic, nutritional, socioeconomic, and cultural factors underscores the significant influence of regional differences on hematological parameters in adolescents. However, northern and eastern Sudan differ in numerous environmental characteristics, including climate, altitude, infectious disease burden, dietary habits, and healthcare access. These regional characteristics could independently influence hemoglobin, leukocyte, and platelet counts.

This study revealed differences in hematological parameters per sex. Females are more likely to have decreasing hemoglobin levels (anemia) and increasing WBC, RDW, and platelets than their male counterparts. Researchers attribute the influence of gender on hematological parameters, including hemoglobin levels, WBC, RDW, and platelets in adolescents, to a combination of biological, hormonal, and physiological factors. Hormonal differences between males and females significantly impact hematological parameters. Testosterone, typically higher in males, promotes erythropoiesis (the production of red blood cells), often resulting in higher hemoglobin and hematocrit levels among males than females (5). Studies have consistently shown that male adolescents have higher hemoglobin concentrations than their female counterparts, particularly during and after puberty (5, 29). Differences in body composition between genders can also influence hematological parameters. Males generally have a higher percentage of lean muscle mass, which can correlate with higher metabolic activity and increased oxygen demand, leading to elevated red blood cell production. In contrast, females may experience fluctuations in hemoglobin levels due to menstrual blood loss, especially during early menstruation (32), which can contribute to lower hemoglobin and RDW values than males. Research indicates that WBC counts can also differ by gender, with females often exhibiting higher WBC counts than males during adolescence. This difference may be attributed to variations in immune responses, with females tending to have stronger responses, which may lead to higher WBC counts (30). Sex differences in platelet counts have also been documented, with some studies indicating that females may have slightly higher platelet counts than males (24, 33). Additionally, RDW, which reflects the variability in red blood cell size, can be influenced by nutritional status and menstrual health in females, potentially leading to variations in RDW values between genders (24). Therefore, sex plays a crucial role in shaping hematological parameters in adolescents through hormonal influences, differences in body composition, and variations in immune function. Understanding these differences is essential for establishing appropriate reference ranges and improving the diagnosis and management of hematological disorders in both male and female adolescents. Further research is needed to better understand the implications of these differences on health outcomes in diverse populations.

In this study, an increase in adolescents’ age was associated with higher hemoglobin levels and lower platelet counts, but not with WBC or RDW. Increasing adolescent age is associated with changes in hematological parameters, particularly hemoglobin levels and platelet counts. These changes can be attributed to various physiological and developmental factors. Previous studies support our findings, as they attributed the association between increasing adolescents' age and higher hemoglobin levels to physiological growth and hormonal influences, while decreasing platelet maturation processes by advancing adolescents' age (29, 34, 35). In our context, this could be explained by children and adolescents of different ages eating the same dish, which can have significant implications for their nutritional status and risk of anemia. When children of different ages eat the same dish, younger children may not always consume sufficient quantities of nutrient-dense foods, potentially increasing their risk of nutritional deficiencies, including anemia (36). As a result, inadequate dietary iron, infections, and socioeconomic status contribute to anemia in young children. These age-related changes underscore the importance of considering developmental factors when evaluating hematological parameters in children and adolescents.

In the current study, although smoking/tobacco use was associated with hemoglobin in univariate linear regression and differed between the two studied regions, it was not associated with hemoglobin in the multivariate linear regression (after adjustment for confounding factors). In contrast, a study conducted by Ahmed et al. reported that smoking affects hematological parameters; smokers had significantly higher hemoglobin levels, WBC, RDW, and PDW compared to the non-smoker group among Sudanese adults (37). Smoking introduces carbon monoxide (CO) into the bloodstream, which binds to hemoglobin more effectively than oxygen, reducing the blood's oxygen-carrying capacity (37–39). This can lead to compensatory mechanisms in the body, such as increased hemoglobin production. However, this increase in hemoglobin may not be effective in improving overall oxygen delivery (38). The lack of association in this study could be explained by the duration and quantity of usage, rather than just asking yes or no questions regarding the use of smoking or tobacco.

The CRP levels were similar between the two studied regions. Existing literature presents contradictory findings regarding the impact of ethnicity on CRP levels (40). This influence appears to depend on the presence or absence of several mediating factors. For instance, Farmer et al. found that ethnicity, gender, and socioeconomic status were each independently associated with baseline CRP levels; however, only socioeconomic status was linked to CRP levels at follow-up (41).

Researchers should view the contradictory data regarding the influence of regional differences, age, and gender on hematological parameters as an incentive to establish their hematological reference ranges specific to their country or region. This study will serve as a foundation for future research aimed at establishing region-specific reference intervals for hematological parameters in Sudan.

### Strengths and limitations of the study

To the best of the authors' knowledge, this is the first comprehensive study to explore the impact of regional differences, age, and gender on hematological parameters and inflammatory biomarkers among adolescents in Sudan. The findings contribute valuable data to the limited research supporting region-specific hematological reference intervals. Furthermore, the results suggest the need for additional research to create national guidelines for hematological parameters to facilitate accurate diagnosis of hematological disorders. However, this study has limitations that should be addressed to improve future research design. It was a cross-sectional study that could not establish causal associations between the variables. Thus, prospective longitudinal studies are necessary to clarify the relationships among the variables studied, hematological parameters, and inflammatory biomarkers in adolescents. Additionally, since the research was conducted in only two regions of Sudan (northern and eastern), the generalizability of the results to all Sudanese adolescents is limited, as potential mediating factors influencing the variables studied cannot be ruled out. The study did not collect information on adolescents' dietary habits or physical activity, which could affect hemoglobin, WBC, platelet, and CRP levels and be influenced by regional differences, age, and gender (42, 43). Several important confounders known to influence hematological parameters were not measured, including nutritional status, BM, pubertal stage, parasitic infections, socioeconomic status, and dietary iron intake. Moreover, information on other factors (e.g., drugs, circadian rhythm) that could influence the reference intervals was not collected. Future studies should incorporate this information to establish region-specific hematological reference intervals that accurately represent all adolescents in Sudan.

## Conclusion

This study identified notable regional variations in hematological parameters among adolescents in Sudan, with northern Sudan showing higher hemoglobin and RDW levels, whereas eastern Sudan had increased WBC and platelet counts. Furthermore, being female and living in eastern Sudan was associated with lower hemoglobin levels, underscoring the necessity for targeted health interventions. Further research is recommended to establish age-, region-, and gender-adjusted cutoff values for hematological parameters in clinical practice to improve the diagnosis and prognosis of hematological disorders among Sudanese adolescents.

## Data Availability

The raw data supporting the conclusions of this article will be made available by the authors, without undue reservation.
